# Peak Fat Oxidation Rate Is Closely Associated With Plasma Free Fatty Acid Concentrations in Women; Similar to Men

**DOI:** 10.3389/fphys.2021.696261

**Published:** 2021-08-02

**Authors:** Jacob Frandsen, Axel Illeris Poggi, Christian Ritz, Steen Larsen, Flemming Dela, Jørn W. Helge

**Affiliations:** ^1^Xlab, Department of Biomedical Sciences, Faculty of Health and Medical Sciences, Center for Healthy Aging, University of Copenhagen, Copenhagen, Denmark; ^2^Department of Nutrition, Exercise, and Sports, Faculty of Science, University of Copenhagen, Copenhagen, Denmark; ^3^Clinical Research Centre, Medical University of Białystok, Białystok, Poland; ^4^Department of Geriatrics, Bispebjerg University Hospital, Copenhagen, Denmark

**Keywords:** fat oxidation rate, fast, repeated exercise, FATmax, substrate availability

## Abstract

**Introduction:** In men, whole body peak fat oxidation (PFO) determined by a graded exercise test is closely tied to plasma free fatty acid (FFA) availability. Men and women exhibit divergent metabolic responses to fasting and exercise, and it remains unknown how the combined fasting and exercise affect substrate utilization in women. We aimed to investigate this, hypothesizing that increased plasma FFA concentrations in women caused by fasting and repeated exercise will increase PFO during exercise. Then, that PFO would be higher in women compared with men (data from a previous study).

**Methods:** On two separate days, 11 young endurance-trained women were investigated, either after an overnight fast (Fast) or 3.5 h after a standardized meal (Fed). On each day, a validated graded exercise protocol (GXT), used to establish PFO by indirect calorimetry, was performed four times separated by 3.5 h of bed rest both in the fasted (Fast) or fed (Fed) state.

**Results:** Peak fat oxidation increased in the fasted state from 11 ± 3 (after an overnight fast, Fast 1) to 16 ± 3 (mean ± SD) mg/min/kg lean body mass (LBM) (after ~22 h fast, Fast 4), and this was highly associated with plasma FFA concentrations, which increased from 404 ± 203 (Fast 1) to 865 ± 210 μmol/L (Fast 4). No increase in PFO was found during the fed condition with repeated exercise. Compared with trained men from a former identical study, we found no sex differences in relative PFO (mg/min/kg LBM) between men and women, in spite of significant differences in plasma FFA concentrations during exercise after fasting.

**Conclusion:** Peak fat oxidation increased with fasting and repeated exercise in trained women, but the relative PFO was similar in young trained men and women, despite major differences in plasma lipid concentrations during graded exercise.

## New and Noteworthy

- Plasma free fatty acid (FFA) concentrations are closely tied to whole body peak fat oxidation rate (PFO) in women.
- PFO relative to lean body mass was similar during repeated exercise in fasting and fed trained women compared with men.
- Plasma FFA concentrations were elevated in women after ~22 h fast compared with men.

## Introduction

The general metabolic response to acute graded exercise is similar across sexes, with increasing fat oxidation rate from light- to moderate-intensity exercise and a decreasing fat oxidation rate from moderate to high-intensity exercise with a concomitant increase in glucose oxidation (Stisen et al., 2006; Vest et al., 2018; Frandsen et al., 2020). When evaluating differences in the oxidation of fat and carbohydrate during exercise between men and women, it is critical to consider a number of variables, i.e., training state, body composition, and hormonal status but also consider the day-to-day reliability in the methodology (Chrzanowski-Smith et al., 2020). Although the majority of studies find that women have a higher fat oxidation rate relative to lean body mass (LBM) during submaximal exercise (Tarnopolsky et al., 1995; Carter et al., 2001; Chenevière et al., 2011), some studies report no differences between men and women (Mittendorfer et al., 2002; Roepstorff et al., 2002).

During exercise, fat is recruited from endogenous and exogenous sources, and there are conflicting results on which fat sources men and women recruit and oxidize during exercise. Most studies find that women, in general, have a higher lipolytic rate [i.e., plasma glycerol and free fatty acid (FFA) rate of appearance] in adipose tissue compared with men (Carter et al., 2001; Nielsen et al., 2003; Mittendorfer et al., 2004, 2009; Mittendorfer, 2005). Yet, there are also studies reporting that women have a higher reliance on intramyocellular lipids (IMCL) and thus lower plasma FFA contribution compared with equally trained men (Roepstorff et al., 2002; Steffensen et al., 2002). A study using ^1^H-MRS found a significant decrease in IMCL after exercise, but to a similar degree in men and women, implying that sex differences are small or even negligible (Roepstorff et al., 2002; Steffensen et al., 2002; White et al., 2003). In a companion study, we found that the plasma FFA availability was closely associated with peak fat oxidation (PFO) in trained men, but whether a similar effect is present in trained women remains uninvestigated (Frandsen et al., 2019). Although several studies (Carter et al., 2001; Horton and Hill, 2001; Mittendorfer et al., 2001; Mittendorfer, 2005; Soeters et al., 2007) have examined the effect of fasting or exercise on the metabolic response in both men and women, it is not known how fasting and repeated exercise affect PFO during a graded exercise test in women.

It is well-known that fasting and repeated exercise both separately increase lipolysis and consequently plasma FFA concentrations in both men and women (Stich et al., 2000). In general, fasting leads to higher plasma FFA concentrations and lower glucose concentrations as an effect of increased lipolytic rate and lower endogenous glucose production in women compared with men (Mittendorfer et al., 2001; Soeters et al., 2007). However, to our knowledge, how the effect of these sex-related metabolic differences to fasting and exercise influences PFO remains unanswered. Thus, the aim of this study was to investigate the response to combined fasting and repeated exercise in trained young women and to analyze and compare these results with previously obtained results in a companion study. We hypothesized that PFO would increase with increasing duration of fasting and prior repeated exercise as a consequence of increased plasma FFA concentrations in women. Then, that PFO relative to LBM would be higher in trained women compared with trained men.

## Methods

### Participants

Twelve healthy endurance-trained women were recruited in this study. This study was approved by the Scientific Ethical Committee of the Greater Region of Copenhagen and adhered to the principles of the Helsinki Declaration (H-19034238). Oral and written information about the nature of this study and the possible risks associated with this study were provided before the women volunteered to participate and signed the consent form. Participants were national age-group or elite-level triathletes or national elite-level in cycling and running. One subject did not complete this study, and for one subject only partial blood data are available due to technical issues with blood sampling.

### General Design

This study design was adopted from a previous study in endurance-trained men (Frandsen et al., 2019).

In brief, participants reported to the laboratory on three occasions. The three visits to the laboratory were separated by >7 days and <14 days. Initially, a brief interview was conducted to inform the participants about the aims and objectives of this study. After this, a dual-energy X-ray absorptiometry (DXA) scan was performed to establish body composition and a $\dot{\text{V}}$O_2max_ test on a cycle ergometer. The two consecutive and randomized visits consisted of four graded exercise tests (GXT) on the same cycle ergometer every fourth hour in either the fed or fasted state. We did not control for the menstrual cycle in this study, but *a posteriori*, we analyzed plasma concentrations of estrogen (Estradiol) and progesterone. We have previously shown that the menstrual cycle phase (i.e., natural physiological fluctuations in sex hormones) does not influence PFO during exercise (Frandsen et al., 2020). All participants were eumenorrheic.

### Experimental Design

#### Pretest Screening

Body composition was determined by a DXA-scan (Lunar Prodigy Advance, Lunar Madison, WI, USA), the $\dot{\text{V}}$O_2max_ test was performed on a cycle ergometer (Monark E839, Värberg, Sweden), and pulmonary gas exchange (indirect calorimetry, Oxycon Pro, Jaeger, Würzburg, Germany) were measured continuously *via* breath by breath. The $\dot{\text{V}}$O_2max_ cycling protocol consisted of a 5-min warm-up period at a workload (WL) of 100 W and after this, the WL increased 25 W/min until volitional exhaustion. The criteria for achieving VO_2_peak were: (i) a leveling off in $\dot{\text{V}}$O_2_ < 2 ml/min/kg body weight, despite a continuous increase in ventilation and WL and (ii) a respiratory exchange ratio (RER) >1.15 during the final 30 s of the test.

#### Fasting Day

The participants arrived at the laboratory after an overnight and non-physical commuting. The first GXT was performed 30 min after the arrival in the laboratory. During the first GXT, 12 ml of blood was sampled during the last 30 s of each WL. Four GXT were performed throughout the day interspersed by 3.5 h of bed rest, i.e., starting every fourth hour. Immediately before the second and third GXT, a 20-ml blood sample was collected, and during the fourth GXT, blood samples were taken as in the first GXT.

#### Fed Day

Before the fed day, the participants were carefully instructed to eat a standardized meal 3 h before arriving at the laboratory. All participants reported having adhered to the instructions. Ten minutes after each GXT in the fed state, a similar standardized meal was provided to the participants. The meal consisted of 80 g of oatmeal [1,235 kilojoules (kJ)], 300 ml of skimmed milk (606 kJ), 30 g of raisins (340 kJ), and 250 ml of chocolate milk (737 kJ), a total of 2,918 kJ (fat = 9.6 E%, carbohydrate = 73.7 E%, and protein = 16.7 E%). The meal was intended to match the energy requirements and was slightly adapted compared with our prior study with a reduction of oatmeal intake from 100 to 80 g to accommodate the differences in resting metabolic rate and fewer WL. Besides the four standardized meals, the fed day was similar to the fast day. A schematic overview of this study design performed in men can be found in the previous study (Frandsen et al., 2019).

### Graded Exercise Tests

Before initiating the graded exercise test, participants rested 5 min on the cycle ergometer to record baseline resting fat oxidation rates. Hereafter, the initial WL was set at 60 W and further increased by 35 W every third minute. Average pulmonary $\dot{\text{V}}$O_2_ and $\dot{\text{V}}$CO_2_ from the last 90 s of every WL were used to calculate the fat oxidation rates (see equation below) and compute a 3rd-degree polynomial regression curve to estimate PFO (highest value on the subsequent non-linear curve). This exercise protocol (GE_35/3_) has previously been validated by Achten et al. (2002) and was terminated when RER was above 1.0 for 30 s. Pulmonary $\dot{\text{V}}$O_2_ and $\dot{\text{V}}$CO_2_ were measured breath by breath with an automated online system (Oxycon Pro, Jaeger, Würzburg, Germany). Before each exercise test, the gas-analyzers and volume sensors were carefully calibrated according to the guidelines of the manufacture. Substrate oxidation was calculated using the equations of Frayn with the assumption that urinary nitrogen excretion, i.e., protein oxidation was negligible (Frayn, 1983):

$$\begin{array}{l} \text{ Fat oxidation }\left(\text{g}/\text{min}\right)\;=\;\left(1.67\times \;\overset{∙}{\text{V}}{\text{O}}_{2}\right)-\left(1.67\times \;\overset{∙}{\text{V}}\text{C}{\text{O}}_{2}\right)\\ \text{Carbohydrate oxidation }\left(\text{g}/\text{min}\right)\;=\;\left(4.55\times \overset{∙}{\text{V}}\text{C}{\text{O}}_{2}\right)-\left(3.21\times \;\overset{∙}{\text{V}}{\text{O}}_{2}\right). \end{array}$$

### Blood Samples

In brief, resting blood samples were collected *via* a Venflon catheter placed in the antecubital vein. A total of 20 ml of venous blood were collected immediately before each GXT, and on the first and fourth GXT (both Fast and Fed), 12 ml of venous blood were collected during the last 60 s of the 3 min at every WL. After collection, blood samples were centrifuged at 4,000 *g* for 10 min at 4°C, and the plasma fraction was immediately stored at −80°C for later analysis (Centrifuge Hettich Universal 30 RF; Hettich, Tuttlingen, Germany). Plasma FFA, glycerol, glucose, lactate, β-hydroxybutyrate, and triacylglycerols (TG) concentrations were analyzed in all blood samples. Basal blood samples were analyzed for insulin, estradiol, and progesterone. All blood samples were analyzed using conventionally commercial assays on a COBAS 6000 analyzer 501C (COBAS Roche, Germany).

### Statistics

Peak fat oxidation during all GXT in women was analyzed by two-way ANOVAs with repeated measures and a Tukey *post-hoc* analysis for multiple comparisons.

Basal blood parameters were analyzed by a mixed-effect model using REML estimation including condition and test number as fixed effects. In case interactions were present, multiple comparison analyses were performed by a Sidak *post-hoc* analysis. Blood samples obtained during exercise were also analyzed by a mixed-effect model including condition (i.e., Fed vs. Fast), test number (e.g., Fed 1 vs. Fed 4), and WL (e.g., 60 W vs. 95 W) as fixed effects. Differences in basal plasma estradiol and progesterone concentration were analyzed by a paired *t*-test. Differences in PFO (mg/min/kg LBM) between women and men were analyzed by a three-way ANOVA including sex, condition, and GXT number with repeated measures and subsequent Tukey *post-hoc* analysis adjusting for multiple comparisons. Furthermore, differences in plasma blood concentrations during exercise were analyzed by a mixed-effect model with fixed effects (type III) for sex (women vs. men), test number (e.g., Fast 1 vs. Fast 4), and WL (e.g., basal vs. 165 W). In case interactions were present, the Tukey *post-hoc* analysis was applied to obtain multiple comparisons. A linear mixed model was conducted to estimate and compare associations between relative PFO (ml/min/kg LBM) and plasma FFA concentrations in women and men. All mixed-effect models included participant-specific random effects to accommodate missing values. Mixed-effect models and ANOVAs were all conducted using SPSS statistics version 25 (IBM software), GraphPad Prism 8.0 (GraphPad Software), and R (R Core Team, 2020). All figures were obtained by using GraphPad Prism 8.0 (GraphPad Software). The significance level was set at *p* < 0.05.

## Results

### Participant Characteristics

The 11 participants were young women (27 ± 4 years), lean (BMI: 22 ± 3 kg/m^2^, fat%: 20.4 ± 5.4), and well-trained ($\dot{\text{V}}$O_2max_: 57.4 ml/min/kg) triathletes, runners, or cyclists (Table 1).

**Table 1:** Characteristics of participants in this study (women) and from a companion study (men).

**Variable**	**Women (*n* = 11)**	**Men (*n* = 10)**
Age, years	27 ± 4	31 ± 6
Height, cm	169 ± 6	183 ± 4^****^
BMI, kg/m^2^	22 ± 3	23 ± 2
Weight, kg	63.9 ± 9.6	77.7 ± 7.5^**^
Lean body mass, kg	48.6 ± 5.8	65.5 ± 5.8^****^
Fat, %	20.4 ± 5.4	12.6 ± 3.6^**^
$\dot{\text{V}}$O_2_max, ml/min	3,610 ± 476	5,079 ± 595^****^
$\dot{\text{V}}$O_2_max, ml/min/kg	57.4 ± 7.0	65.9 ± 6.1^**^
$\dot{\text{V}}$O_2_max, ml/min/kg LBM	74.8 ± 9.1	77.6 ± 6.4

*Anthropometric characteristics. BMI, body mass index. n = 21*,

** *p < 0.01*,

**** *p < 0.0001. Data are represented as mean ± SD*.

### Fat Oxidation

**Figure 1:**
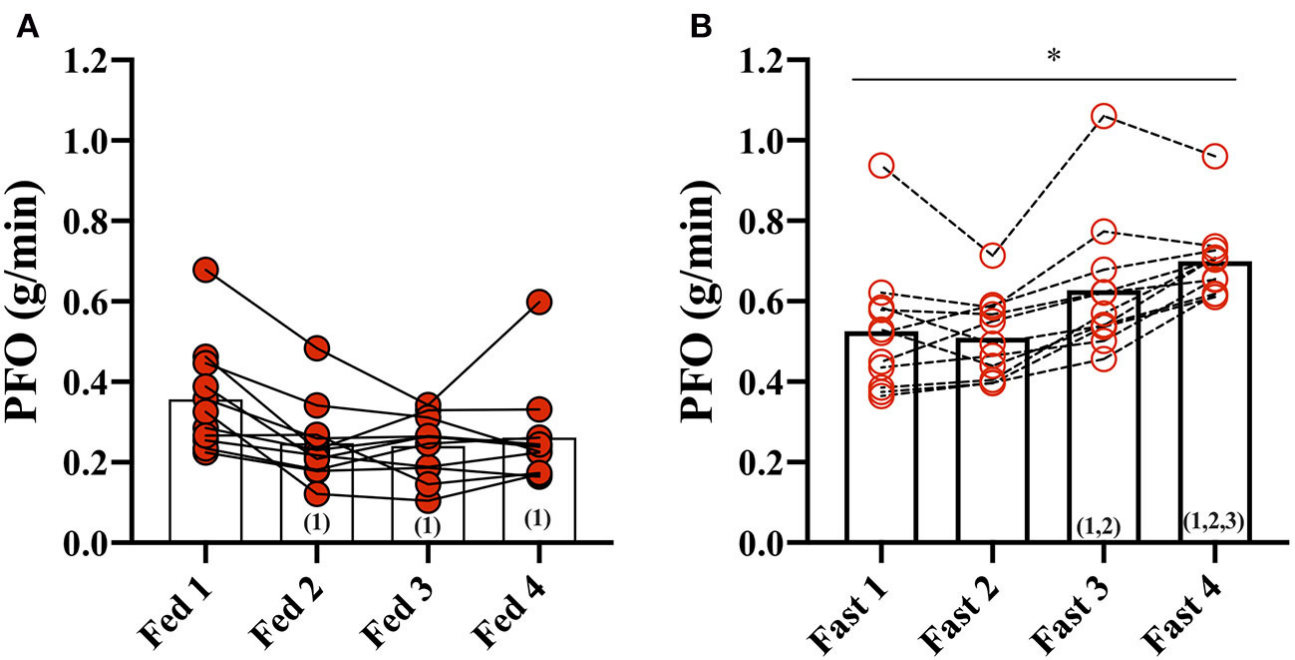
Peak fat oxidation (PFO) during exercise in the fasted and fed state. **(A)** PFO (g/min) during GXT: Fed 1, 2, 3, and 4 and **(B)** during GXT Fast 1, 2, 3, and 4. * represents the significant difference between conditions (Fed vs. Fast) within the same test number (e.g., GXT 1 vs. 2) and (number) represents the statistical difference from GXT number.

In the Fed state (3.5 h after a meal) (Fed 1), PFO was 0.36 ± 0.13 g/min but decreased to 0.25 ± 0.10 g/min after the second fed test (Fed 2) and remained unchanged throughout the fed days (Fed 2, 3, and 4) (Figure 1). Fat oxidation was higher during all GXT in the fasted state compared with the fed state, and Fast 3 (0.63 ± 0.17 g/min) and Fast 4 (0.70 ± 0.10 g/min) were increased compared with the first test in the fasted state (Fast 1).

### Basal Blood Samples

We found a significant interaction between Fast vs. Fed and GXT on plasma glucose concentration; however, multiple comparison analyses revealed no significant differences (Table 2). Plasma FFA concentrations were significantly higher at baseline before every GXT in the fasted state compared with the fed state where it remained unchanged, whereas it increased in the fasted state from first (Fast 1) to the third and fourth (Fast 3 and 4, *post-hoc p* < 0.0001). At baseline before the first test (Fast 1 vs. Fed 1), plasma glycerol concentrations were similar, but during GXT 2, 3, and 4, plasma glycerol concentrations were higher in the fast compared with the fed state (interaction: *p* = 0.002; Table 2). Plasma ketone concentrations (β-hydroxybutyrate) increased from the Fast 1 test to Fast 3 and Fast 4 (main effect: test number *p* < 0.0001) and remained chronically suppressed in the fed states (Fed 1–4). The plasma TG concentration was increased before Fed 4 compared with Fed 1 (*post-hoc*: *p* = 0.001). Plasma insulin concentrations were significantly increased in the fed condition compared with fast (Fed vs. Fast, *p* = 0.0005) in all four GXT (Table 2).

**Table 2:** Basal blood plasma concentrations obtained before each exercise test.

	**GXT1**		**GXT2**		**GXT3**		**GXT4**		**Mixed model analysis**	
**Variable**	**Fast**	**Fed**	**Fast**	**Fed**	**Fast**	**Fed**	**Fast**	**Fed**	**Main effect: condition or test, (*p*-value)**	**Interaction: condition × test (*p*-value)**
Glucose (mmol/l)	5.1 ± 0.7	4.7 ± 0.7	4.9 ± 0.4	5.1 ± 0.3	4.8 ± 0.5	5.0 ± 0–4	4.6 ± 0.4	5.0 ± 0.4	*ns*.	=0.0074
FFA (μmol/l)	404 ± 203	170 ± 186^**^	511 ± 145	74 ± 61^****^	707 ± 236^ΔΔΔΔ^	68 ± 41^****^	865 ± 210^ΔΔΔΔ^	115 ± 110^****^	Condition: <0.0001, test: =0.0005	<0.0001
Glycerol (μmol/l)	61 ± 38	49 ± 31	69 ± 33	21 ± 10^Δ^^***^	84 ± 35	22 ± 13^Δ^^****^	88 ± 33	26 ± 17^****^	condition: <0.0001, test: *ns*.	=0.0015
β-hydroxybutarate (mmol/l)	0.09 ± 0.08	0.05 ± 0.06	0.12 ± 0.08	0.04 ± 0.02	0.28 ± 0.19^ΔΔΔΔ^	0.03 ± 0.01^****^	0.59 ± 0.30^ΔΔΔΔ^	0.04 ± 0.02^****^	Condition: =0.0002, test: <0.0001	<0.0001
lactate (mmol/l)	0.73 ± 0.16	1.05 ± 0.42	0.66 ± 0.18	0.82 ± 0.16	0.72 ± 0.18	0.76 ± 0.16	0.77 ± 0.15	0.84 ± 0.17	Condition: =0.032, test: =0.029	*ns*.
Triglyceride (mmol/l)	0.66 ± 0.19	0.73 ± 0.35	0.55 ± 0.21^Δ^	0.71 ± 0.30	0.57 ± 0.22	0.82 ± 0.29^**^	0.56 ± 0.19	0.90 ± 0.32^****^^ΔΔ^	Condition: = 0.032, test: =0.009	=0.0002
Insulin (μmol/l)	29 ± 7	54 ± 41^*^	23 ± 6	51 ± 22^**^	22 ± 8	53 ± 22^**^	20 ± 6	57 ± 22^***^	Condition: =0.0005, test: *ns*.	*ns*.

*GXT, graded exercise test; FFA, free fatty acids; ns, not significant. Post-hoc analysis: Fast within similar GXT*,

* *p < 0.05*,

** *p < 0.01*,

*** *p < 0.001*,

**** *p < 0.0001, GXT1 within similar condition*,

Δ *p < 0.05*,

ΔΔ *p <0.01*,

ΔΔΔΔ *p < 0.0001. Data are represented as mean ± SD*.

**Figure 2:**
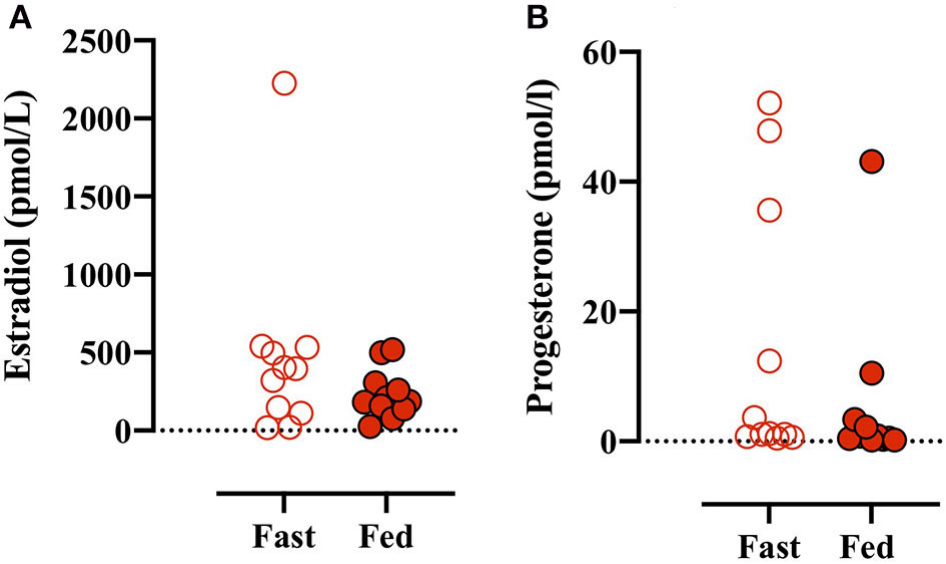
**(A)** Plasma estradiol concentrations measured before the first GXT on the Fast (open red circles) and Fed (closed red circles) days. **(B)** Plasma progesterone concentrations before the initial GXT on the Fast and Fed days. Paired *t*-test (estradiol): *p* = 0.238 and (progesterone): *p* = 0.288.

Plasma estradiol and progesterone were measured in all plasma baseline blood samples. There was no main effect between conditions (i.e., Fed vs. Fast), but there was an effect of test number (e.g., GXT 1 vs. 4) (data not shown). A simple analysis (i.e., paired Student's *t*-test) found no difference between estradiol concentrations before the Fast day compared with the Fed day (Figures 2A,B). Plasma progesterone concentrations were despite numerically large interindividual and intraindividual variations not different between conditions or different tests throughout the day (data not shown). Based on sex hormone concentration reference values (Stricker et al., 2011), four participants were in the luteal phase [from +2 to +14 days after luteinizing hormone (LH) peak] and seven participants were in the follicular phase (−15 to 0 days from LH peak) before the fed day (Figures 2A,B). Similarly, four participants were in the luteal phase, six participants in the follicular phase, but one participant was in the late-follicular phase with very high plasma estrogen concentrations (2,204 ± 89 pmol/L) before the fast day (Figure 2A).

### Plasma Metabolites and Substrates During Exercise

**Figure 3:**
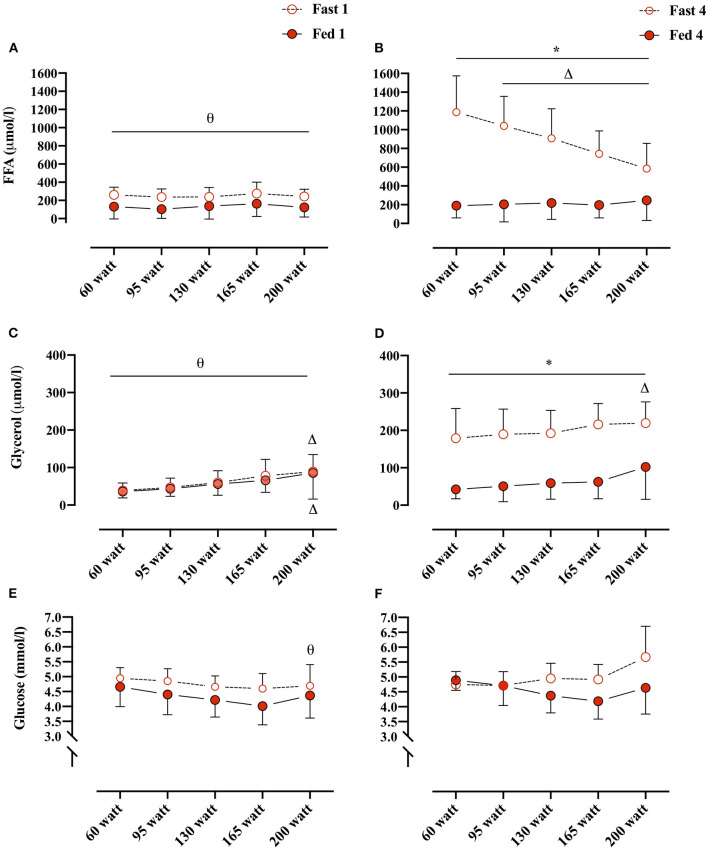
**(A,B)** Plasma FFA concentrations (μmol/L) at, 60, 95, 130, 165, and 200 W during Fed 1 (open red circles), Fast 1 (closed red circles) and Fed 4 (open red circles), Fast 4 (closed red circles), respectively. **(C,D)** Plasma glycerol concentrations (μmol/L)/workload (W) during Fed 1, Fast 1 and Fed 4, Fast 4, respectively. **(E,F)** Plasma glucose concentrations (mmol/L)/workload (WL) (W) during Fed 1, Fast 1 and Fed 4, Fast 4, respectively. *, difference between condition within test number and WL; Δ, difference from 60 W within test number and condition; θ, difference between test number within condition and WL. 60 W: Fast 1, *n* = 10, Fed 1, *n* = 11; 95 W: Fast 1, *n* = 10, Fed 1, *n* = 11; 130 W: Fast 1, *n* = 9, Fed 1, *n* = 11; 165 W: Fast 1, *n* = 10, Fed 1, *n* = 9; 200 W: Fast 1, *n* = 7, Fed 1, *n* = 6. 60 W: Fast 4, *n* = 9, Fed 4, *n* = 8; 95 W: Fast 4, *n* = 10, Fed 4 and 8; 130 W: Fast 4, *n* = 9, Fed 4, *n* = 8; 165 W: Fast 4, *n* = 10, Fast 4, *n* = 7; 200 W: Fast 4, *n* = 7, Fed 4, *n* = 6. Data are presented as mean ± SD (error bars).

Plasma FFA concentrations changed overall as an effect of both conditions (i.e., Fed vs. Fast), test number (i.e., GXT 1 vs. 4), and WL (e.g., 60 W vs. 160 W) (REML, type III, interaction: WL × condition × test number, *p* < 0.0001). Plasma FFA concentrations were not different between Fed 1 and Fast 1 at any of the WL and also not different between Fed 1 and Fed 4 at any WL. However, in the fasted state, plasma FFA concentrations were significantly elevated at every WL in Fast 4 compared with Fast 1 (Figures 3A,B). In the Fast 4 test, plasma FFA concentrations were decreased from 60 W through the remaining WL and were elevated compared with all WL in the Fed 4 test (Figures 3A,B). Plasma glycerol concentrations were not different between the first tests (i.e., Fast 1 vs. Fed 1) at any WL (Figures 3C,D). Similar to plasma FFA, plasma glycerol concentrations were elevated at every WL in Fast 4 compared with Fed 4 and also with Fast 1 (Figures 3C,D). In the fed state, plasma glycerol concentrations remained unchanged throughout GXT (Figures 3C,D). Plasma glucose concentrations were not different between conditions (i.e., main effect: condition, *p* = 0.084). However, multiple comparison analyses showed a significant difference between Fast 1 and Fast 4 at 200 W (Figures 3E,F).

## Differences Between Sexes

Subsequently, we compared the data from the women in this study with the published data from trained men obtained with a completely identical protocol (Frandsen et al., 2019). The initial WL in the previous study was 95 W, whereas, in this study, the initial WL was set at 60 W in order to accommodate the lower absolute $\dot{\text{V}}$O_2max_ and to obtain a sufficient number of data points. Both the protocols were thereafter set with an increase of 35 W every third minute. Subsequently, the workloads are termed as WL.

### Participant Characteristics

Women had a lower LBM (kg) and $\dot{\text{V}}$O_2max_ (ml/min and ml/min/kg) but higher fat mass (%), compared with men. However, age, BMI, and $\dot{\text{V}}$O_2max_ normalized to LBM (ml/min kg LBM) were similar between the groups (Table 1, Frandsen et al., 2019).

### Fat Oxidation

**Figure 4:**
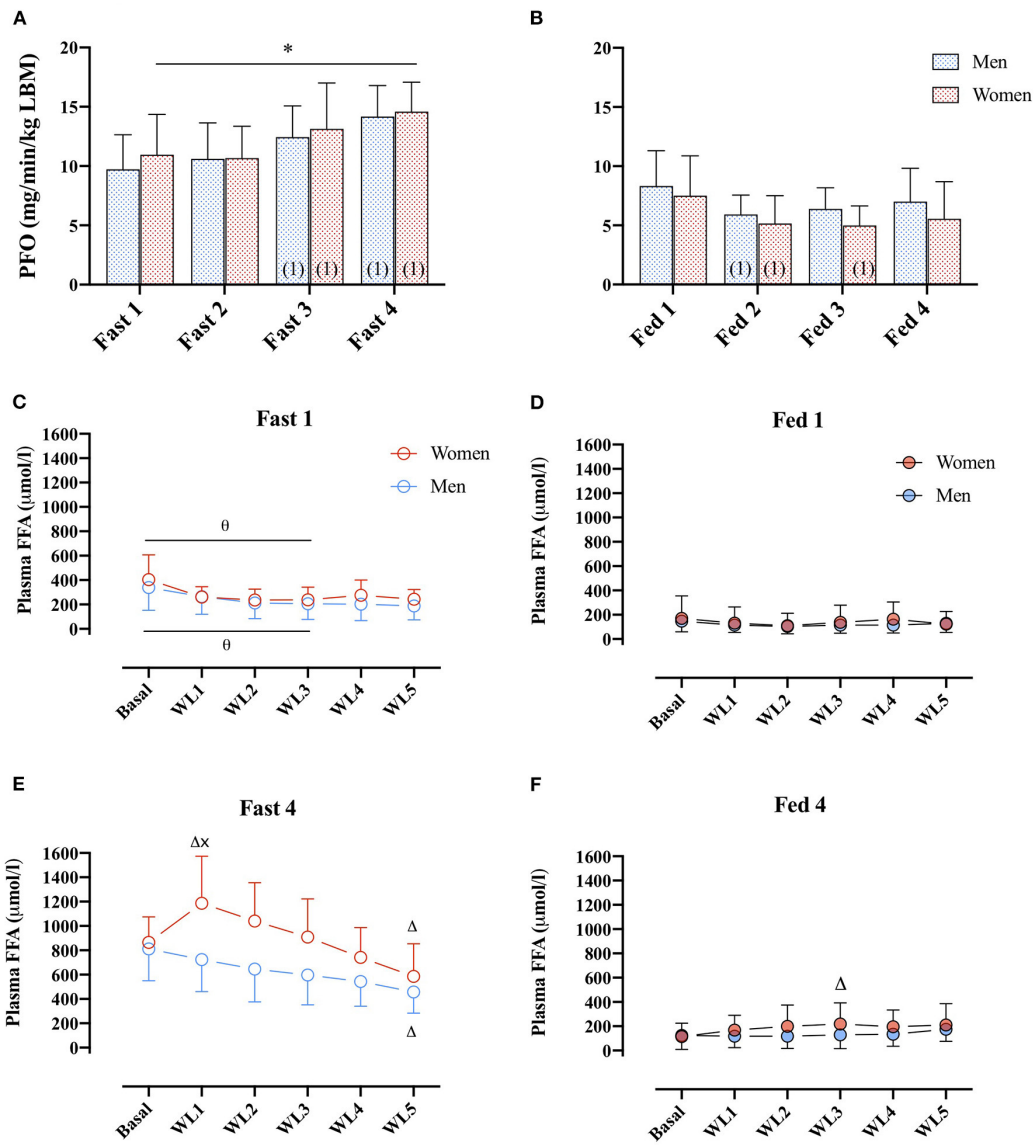
**(A)** PFO (mg/min/kg FFM) during GXT Fast 1, 2, 3, and 4 in men (dashed blue bars) and women (dashed red bars). (number) represents statistical difference from GXT number. **(B)** PFO (ml/min/kg FFM) during GXT Fed 1, 2, 3, and 4 in men and women.* represents the significant difference between conditions within similar GXT, (1) represents the significant difference from first GXT within similar condition. **(C,D)** Plasma FFA concentrations (μmol/L) during GXT Fast 1, Fed 1, respectively, at rest (basal) and at WL 1, 2, 3, 4, and 5, representing 60, 95, 130, 165, and 200 W in women and 95, 130, 165, 200, and 235 W in men. **(E,F)** Plasma FFA concentrations (μmol/L) during Fast 4 and Fed 4, respectively. Δ represents the significant difference from Basal within same GXT and sex, x represents the significant difference between sex within same GXT and WL, and θ represents the significant difference between GXT within similar WL and sex. Women Fast 1: Basal, *n* = 11, WL1, *n* = 9, WL2, *n* = 10, WL3, *n* = 9, WL 4, *n* = 10, WL5, *n* = 7; Women Fast 4: basal, *n* = 10, WL1, *n* = 9, WL2, *n* = 10, WL3, *n* = 9, WL4, *n* = 10, WL5, *n* = 7. Men Fast 1: basal, *n* = 10, WL1, *n* = 9, WL2, *n* = 9, WL3, *n* = 9, WL4, *n* = 9, WL5, *n* = 9. Men Fast 4: basal, *n* = 10, WL1, *n* = 9, WL2, *n* = 9, WL3, *n* = 9, WL4, *n* = 9, WL5, *n* = 9. Data are represented as mean ± SD.

No differences in PFO (ml/min/kg LBM) between men and women were found (main effect: sex, *p* = 0.794). However, in the women, PFO (ml/min/kg LBM) was higher in the fasted compared with the fed state in all four GXTs (Figures 4A,B), whereas in men, this difference was only present from the second GXT onward.

### Plasma Metabolites and Substrates

During Fast 1, there was no difference in plasma FFA concentration between men and women (Figure 4C). In both men and women, plasma FFA concentrations were elevated in the basal state and the first three WL in Fast 4 compared with Fast 1. Furthermore, in Fast 4, (Figure 4E), basal plasma FFA concentrations were similar in women and men, respectively (865 ± 210 vs. 811 ± 261 μmol/L, *p* > 0.99), but during the last 60 s of the first WL (WL1), plasma FFA concentrations were 43% different (1,186 ± 387 and 768 ± 285 μmol/L in women and men, *post-hoc*: *p* = 0.006; Figure 4E). In women, there was a significant increase from baseline to WL1. At the last WL (WL5), plasma FFA concentrations were decreased in both women and men compared with baseline (Figure 4E). In the Fed state, there were no differences in plasma FFA concentrations between women and men, or Fed 1 or 4 were found (main effect: sex, *p* = 0.78, Figures 4D,F).

Plasma glycerol concentrations were similar between men and women at all WL after an overnight fast (Fast 1) (data not shown). In women, plasma glycerol concentrations were higher compared with men at all WL during Fast 4 (data not shown). In the fed state (both Fed 1 and Fed 4), plasma glycerol concentrations were not different between men and women (main effect: sex, *p* = 0.93, data not shown).

### Association Between Plasma FFA Availability and PFO

**Figure 5:**
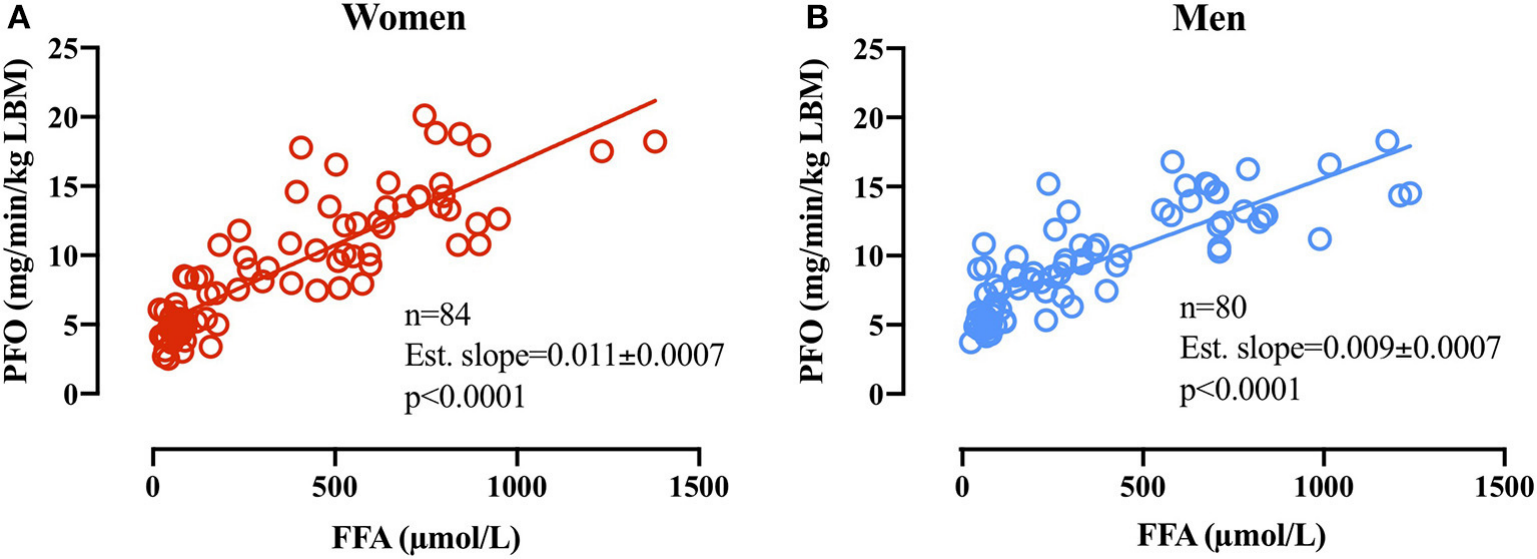
**(A)** Relative PFO (mg/min/kg FFM) plotted against basal plasma FFA concentrations (μmol/L) before subsequent GXT in women **(A,B)** men. Due to technical issues, four basal blood samples were not obtained in the women (84 basal blood samples).

Linear regression analysis on PFO (mg/min/kg LBM) plotted against the associated basal plasma FFA concentration in women (*n* = 84) revealed an estimated slope of 0.011 ± 0.0007 (*p* < 0.0001 different from 0) and in men revealed an estimated slope of 0.009 ± 0.0007 (*p* < 0.0001 different from 0). The difference in slopes between men and women was significant (*p* = 0.025; Figure 5).

## Discussion

### Main Findings

The main finding was that PFO increases concomitant with increasing plasma FFA concentrations in trained women. Furthermore, based on the data from a prior published study in men, we found no difference between men and women in relative PFO (ml/min/kg LBM) either in the fasted or the fed state. A similar PFO in women and men was present in spite of significant differences in plasma concentrations of FFA and glycerol after combined fasting and graded exercise.

### The Effect of Fasting and Exercise on Substrate Availability and Oxidation in Women

Compared with the fed state, PFO was higher during all GXT in the fasted state, reflected in elevated plasma FFA concentrations and lowered insulin concentrations but similar glucose concentrations. It is well-known that fasting exerts a potent metabolic response in men (Mittendorfer et al., 2001), but very few studies have investigated the combined effect of fasting and exercise and the relationship with PFO (Frandsen et al., 2019). Plasma FFA concentrations increased >2-fold from the first to the last test in the fasted state. Interestingly, despite this increase in plasma FFA, glycerol concentrations were not significantly different between the tests. As venous plasma concentrations reflect the balance between substrate delivery to the plasma and tissue uptake, it may indicate that either (i) adipose tissue TG were incompletely degraded or (ii) there is disproportional skeletal muscle and/or (iii) liver tissue uptake (glycerol to *de novo* glucogenesis) (Coppack et al., 1999). It has previously been found that from 14 to 22 h of fasting, the increase in glycerol Ra was smaller in women compared with men, despite similar plasma FFA concentrations. This finding could be a consequence of sex differences in plasma epinephrine concentrations and the adipose tissue sensitivity toward epinephrine (Mittendorfer et al., 2001). Yet, in spite of unchanged lipolytic rate (i.e., plasma glycerol concentration) from 10 to 22 h of fasting in women, our present data show that in women, the plasma FFA concentrations are increased in a dose-dependent manner with fasting and repeated graded exercise and that this does influence PFO.

Several studies have used isotopically labeled tracer methodology to investigate the fat and glucose kinetics during exercise but with conflicting results (Carter et al., 2001; Mittendorfer et al., 2002; Roepstorff et al., 2002; Steffensen et al., 2002). It has been reported that compared with men, women have an increased oxidation of IMCL and a reciprocal lower plasma FFA oxidation relative to the energy requirement (Romijn et al., 2000; Roepstorff et al., 2002; Steffensen et al., 2002). Contrarily, it has also been reported that women rely more on plasma FFA as fuel during submaximal exercise (Friedlander et al., 1998; Mittendorfer et al., 2002). This current data illustrate that during fasting and after repeated graded exercise, plasma FFA remains an important substrate regulating PFO during graded exercise in women. A significant decrease in plasma FFA concentrations from the first WL (60 W) and the rest of the WL in Fast 4 indicates an enhanced skeletal muscle tissue uptake relative to the lipolytic rate (Figure 3B). The fate of the plasma FFA, whether immediately oxidized or stored as IMCL, cannot be decided by the current applied methodology. In women, it has been demonstrated that, during exercise until 65% of $\dot{\text{V}}$O_2max_, the absolute amount of plasma FFA oxidized (FFA Rd) remains similar to the oxidation rate at 25% of $\dot{\text{V}}$O_2max_, but that the IMCL oxidation increases from 25 to 65% of $\dot{\text{V}}$O_2max_ (Romijn et al., 2000). Additionally, at 85% of $\dot{\text{V}}$O_2max_, the plasma FFA oxidation significantly decreases (Friedlander et al., 1998; Romijn et al., 2000). As the plasma glycerol concentrations (i.e., proxy marker of lipolysis) during Fast 4 is clearly elevated from both Fed 1 and Fast 1, there is less doubt that the lipolytic rate is markedly elevated (Figures 3C,D). The decreasing plasma FFA concentrations during Fast 4 together with a potential similar oxidation rate from 25 to 65% $\dot{\text{V}}$O_2max_ (Romijn et al., 2000) (e.g., 60 to 135 W) may therefore be a result of decreased FFA release from adipose tissue and/or increased non-oxidative storage within the myocyte.

### Sex Differences in PFO in the Fasted and Fed State

Despite differences in body composition, $\dot{\text{V}}$O_2max_ (ml/min/kg LBM) were not different (Students *t*, unpaired, *p* = 0.29; Table 1) between the men and women, which implies that their training status was comparable.

It is a recurrent finding that women rely more on fat oxidation during submaximal exercise than men (Horton et al., 1998; Carter et al., 2001; Venables et al., 2004; Chenevière et al., 2011). Furthermore, it is often recognized that women have a higher adipose tissue lipolytic rate both at rest and during exercise (Carter et al., 2001; Mittendorfer et al., 2002; Soeters et al., 2007; Tarnopolsky, 2008). Yet, not all studies are unanimous, and similar fat oxidation rates during different exercise intensities have also been found between sexes (Romijn et al., 2000; Mittendorfer et al., 2002; Roepstorff et al., 2002). Herein, we found that PFO was not different between trained young women and men (Frandsen et al., 2019), either when a standardized meal was received 3.5 h prior to the exercise bout or when fasted in either ~10, 14, 18, or 22 h. Fasting in men and women has been evaluated previously, but to the best of our knowledge, not in combination with repeated exercise. As previously cited (Mittendorfer et al., 2001), it has been found that women after a 14 h fast (overnight) have a higher glycerol Ra (lipolytic rate) and plasma FFA concentrations compared with men, but that after 22 h of fast, the sex-difference is diminished, despite a lower plasma insulin concentration in the women. It should be mentioned that Mittendorfer et al. did not measure fat oxidation rates and, in contrast to our study, matched men and women according to relative fat mass (% BW: 23, 24% men and women, respectively), thus comparisons should be done with caution.

### Sex Differences in Substrate Availability and the Relations to PFO

From basal to the first exercise WL (WL1; 60 W and 95 W) in men and women, respectively, plasma FFA concentrations increased in women and were significantly higher than in men (Fast 4, Figure 4E). This was further reflected in plasma glycerol concentrations that increased from basal to the remaining WL and to a higher concentration compared with men in the first three WL. The increased plasma glycerol concentrations are in line with previous findings of a higher lipolytic rate in women compared with men. However, the sharp rise in plasma FFA concentrations from basal to WL1 (Figure 4E) is somewhat intriguing. Differences in plasma FFA concentration in response to walking between men and women have previously been reported, but the mechanism remains elusive (Blatchford et al., 1985). It may be that higher lipolysis in a larger adipose tissue mass (fat mass: 13 ± 5 kg vs. 8 ± 4 kg, *p* = 0.02, women and men, respectively) and a smaller skeletal muscle mass (LBM: 49 ± 6 kg vs. 66 ± 6 kg, *p* < 0.0001, women and men, respectively) to absorb the plasma FFA in women can explain this difference. It is possible that our results show an overshoot in lipolytic activity relative to the skeletal muscle FFA uptake capacity in women (WL1) but not in men after 22 h of fasting and repeated graded exercise. Despite this marked increase in plasma FFA from rest to WL1 (Fast 4) in women, the linear regression analysis of basal plasma FFA concentrations and PFO in the following GXT demonstrates a close relationship between plasma FFA availability and PFO in women and men. The estimated slopes (i.e., 0.011 ± 0.0007 and 0.009 ± 0.0007 in women and men, respectively), show that an arbitrary increase of 500 μmol/L in plasma FFA in women would increase PFO with 5.5 mg/min/kg LBM, whereas in men, a similar increase in FFA concentrations would increase PFO with 4.5 mg/min/kg LBM. Despite the modest difference, it does seem to indicate that women rely more on plasma FFA as a substrate when plasma FFA concentrations are manipulated. Additional research into the differences in lipid kinetics in women and men during exercise in the fasted state (< overnight fast) is warranted.

The simple methodological design in this study does not allow the analysis of the origin (i.e., endogenous/exogenous) of the substrates oxidized after fasting and a standardized meal during exercise. Furthermore, we did not control for the menstrual cycle phase in the women attending this study. Studies have found that estrogen may influence the substrate metabolism; however, we have previously found that physiological relevant fluctuations in estrogen and progesterone concentrations do not influence PFO measured after an overnight fast and a similar exercise protocol (Frandsen et al., 2020). Also, we assumed that protein oxidation is negligible and constant, which most likely is the case under short-term fasting and in the fed state; however, the extent to which protein oxidation is significantly increased during a GXT after 20 h of fasting is uncertain but would be worthwhile to investigate further. Despite the identical methodological approaches, the companion study in men was conducted 2 years previously, and this design was adopted to women, i.e., the initial WL in the GXT and the energy content of the standardized meal were different. Yet, the same apparatus was used, and internal controls were conducted on blood analysis to ensure comparable results.

### Conclusion

First, we found that women increase PFO in close relationship with increases in plasma FFA concentrations during continuous fasting and repeated graded exercise bouts. Underlining the importance of careful standardization prior to testing PFO with regards to fasting time and physical activity. Second, we found no difference in PFO relative to LBM when comparing trained women with trained men despite a difference in plasma FFA availability.

## Data Availability Statement

The raw data supporting the conclusions of this article will be made available by the authors, without undue reservation.

## Ethics Statement

The studies involving human participants were reviewed and approved by Ethical Committee of Greater Copenhagen Region. The patients/participants provided their written informed consent to participate in this study.

## Author Contributions

JF and JWH conceived and designed research. JF and AP performed experiments. JF and JWH analyzed data. JF interpreted results of experiments, prepared figures, and drafted manuscript. JF, AP, CR, SL, FD, and JWH edited and revised manuscript and approved final version of manuscript. All authors contributed to the article and approved the submitted version.

## Conflict of Interest

The authors declare that the research was conducted in the absence of any commercial or financial relationships that could be construed as a potential conflict of interest.

## Publisher's Note

All claims expressed in this article are solely those of the authors and do not necessarily represent those of their affiliated organizations, or those of the publisher, the editors and the reviewers. Any product that may be evaluated in this article, or claim that may be made by its manufacturer, is not guaranteed or endorsed by the publisher.
